# *Acinonyx jubatus*-Inspired Quadruped Robotics: Integrating Neural Oscillators for Enhanced Locomotion Control

**DOI:** 10.3390/biomimetics9060318

**Published:** 2024-05-27

**Authors:** Eric Alberto Hernández-Flores, Yazmín Mariela Hernández-Rodríguez, Rosario Munguía-Fuentes, Rafael Bayareh-Mancilla, Oscar Eduardo Cigarroa-Mayorga

**Affiliations:** 1 Sistemas Autónomos de Navegación Aérea y Submarina (SANAS), Unidad Mixta Internacional (UMI), Centro de Investigación y de Estudios Avanzados del IPN, Av. Instituto Politécnico Nacional 2508, Col. San Pedro Zacatenco, Gustavo A. Madero, Ciudad de México 07360, Mexico; eric.hernandezf@cinvestav.mx; 2 Unidad Profesional Interdisciplinaria en Ingeniería y Tecnologías Avanzadas del Instituto Politécnico Nacional (UPIITA-IPN), Av. Instituto Politécnico Nacional 2580, Col. San Pedro Zacatenco, Gustavo A. Madero, Ciudad de México 07360, Mexico; yazmin.hernandez@cinvestav.mx (Y.M.H.-R.); mrmunguiaf@ipn.mx (R.M.-F.); ocigarroam@ipn.mx (O.E.C.-M.)

**Keywords:** quadruped robot, neural oscillators, CPG, Multi-Gait Locomotion, *Acinonyx jubatus*

## Abstract

This study presents the design, simulation, and prototype creation of a quadruped robot inspired by the *Acinonyx jubatus* (cheetah), specifically designed to replicate its distinctive walking, trotting, and galloping locomotion patterns. Following a detailed examination of the cheetah’s skeletal muscle anatomy and biomechanics, a simplified model of the robot with 12 degrees of freedom was conducted. The mathematical transformation hierarchy model was established, and direct kinematics were simulated. A bio-inspired control approach was introduced, employing a Central Pattern Generator model based on Wilson–Cowan neural oscillators for each limb, interconnected by synaptic weights. This approach assisted in the simulation of oscillatory signals for relative phases corresponding to four distinct gaits in a system-level simulation platform. The design phase was conducted using CAD software (SolidWorks 2018), resulting in a 1:3-scale robot mirroring the cheetah’s actual proportions. Movement simulations were performed in a virtual mechanics software environment, leading to the construction of a prototype measuring 35.5 cm in length, 21 cm in width, 27 cm in height (when standing), and weighing approximately 2.1 kg. The experimental validation of the prototype’s limb angular positions and trajectories was achieved through the image processing of video-recorded movements, demonstrating a high correlation (0.9025 to 0.9560) in joint angular positions, except for the knee joint, where a correlation of 0.7071 was noted. This comprehensive approach from theoretical analysis to practical implementation showcases the potential of bio-inspired robotics in emulating complex biological locomotion.

## 1. Introduction

Achieving high-speed locomotion in robots poses a significant challenge in robotics, requiring mechanical designs that combine the necessary speed and strength for flight phases [1]. This phase, crucial for achieving high speeds, demands not only sophisticated design and materials but also advanced control systems to manage dynamic balance and propulsion. The development of robots that mimic the biomechanics of real animals has garnered considerable interest in recent years, driven by their potential applications. Despite the vast number of robots designed globally, only a small proportion of quadruped robots have been constructed with a focus on tasks requiring running or galloping at high speeds. The endeavor to develop robots that can mimic the biomechanics of real animals has attracted significant interest, particularly over recent years. This surge in interest is largely attributed to the diverse potential applications of such robots, ranging from search and rescue operations in hazardous environments to exploratory missions in uncharted territories, where the agility and versatility of animal-like locomotion could offer unparalleled advantages [2,3].

Despite the global efforts to design and innovate within the robotics community, the creation of quadruped robots capable of executing tasks such as running or galloping at high speeds remains relatively rare. This scarcity is partly due to the complex interplay of factors required to replicate the efficient, high-speed locomotion observed in nature. Animals that excel in rapid movement, such as cheetahs or horses, have evolved highly specialized anatomical features and muscle structures that enable their impressive speed and agility. Replicating these features in robotic systems involves overcoming significant engineering challenges, including the development of lightweight yet robust materials, precise actuators, and sophisticated algorithms for motion planning and control [3,4]. Furthermore, high-speed locomotion in robots also requires advanced perception systems to navigate and adapt to varying terrains in real-time, a necessity for maintaining balance and direction at high velocities. The integration of these systems into a cohesive unit that can perform complex maneuvers at high speeds necessitates a multidisciplinary approach, drawing on insights from biomechanics, materials science, mechanical engineering, and computer science [5].

Continued advancements in technology and design methodology are gradually closing the gap between robotic capabilities and the natural world’s benchmarks, setting the stage for a future where robots can move with the grace, speed, and agility of their biological counterparts. The evolution of quadruped robots designed for high-speed locomotion has been marked by significant advancements over the past two decades, as resumed in Table 1. These developments, ranging from early prototypes with articulated legs and energy recoil systems to sophisticated models employing biarticular muscle concepts and Central Pattern Generators (CPGs), highlight the interdisciplinary efforts to mimic the efficient and agile movements of animals such as *Acinonyx jubatus*. Table 1 outlines key milestones in the field, including innovations in mechanical design, control strategies, and the implementation of neurobiologically inspired systems, illustrating the progress from Ohio State University’s initial attempts in 2001 to the remarkable speeds achieved by Boston Dynamics’ Wildcat in 2012 and the lightweight efficiency of the EPFL’s Cheetah-cub in 2013. Each entry in this table not only underscores the technical achievements but also points towards the increasing complexity and capability of quadruped robots in navigating and performing tasks in varied terrains at high speeds.

As shown in Table 1, the development of quadruped robots has been an area of active research. The aim of such studies is focused on the reliable traversal of challenging, irregular terrain, which is beyond the capabilities of traditional wheeled mobile robots. Recent advancements in the design of high-performance actuators and hardware structures have led to developments and research in algorithms that mimic the movement patterns of quadrupeds. Table 2 presents a summary of a study on the application of CPGs applied to legged locomotion [12], exploring various techniques for pattern generation to emulate natural movements and overcome obstacles. It highlights the diverse applications of CPGs, from simple movement generation to more complex motion control involving feedback mechanisms and trajectory generation. The citations indicate the range of contributions made in these areas over the years, relating to both quadrupeds and CPG implementation.

The exploration of CPGs to model biomimetic locomotion in quadrupeds has seen an increase in studies tailored to bipedal, quadrupedal, and morphologically diverse reconfigurable systems. However, despite the advances made in the field, there remains an opportunity to refine CPG models to achieve more accurate mimetics of biological movement. The selection of CPGs, particularly those based on Wilson–Cowan neural networks, for the design of a quadruped robot inspired by the *Acinonyx jubatus* is rooted in their demonstrated efficacy in replicating the nuanced patterns of animal gait, particularly in quadrupeds [18,19]. This preference is affirmed when compared to other neural control systems, like those based on reinforcement learning or artificial neural networks, which, although potent, might not naturally synchronize the inter-limb coordination required for steady reliable locomotion over a variety of terrains [3,20]. The Wilson–Cowan model, with its foundation in biological and theoretical neuroscience, provides a robust framework for capturing the continuous interplay of excitatory and inhibitory neural dynamics that characterize animal locomotion [2,20,21]. This model’s adaptability is especially pertinent when programming the locomotion for terrains that demand high adaptability and fault tolerance, paralleling the capabilities of the *Acinonyx jubatus*, which must adjust its gait and posture dynamically to maintain stability and speed. This work presents the design and development of a robot inspired by the *Acinonyx jubatus*, applying a CPG based on Wilson–Cowan neural networks as introduced in [18]. This study emphasizes the potential of CPGs to streamline the movement dynamics of robotic systems, further advancing the field by integrating complex neural network models for more agile and life-like robotic locomotion. By drawing inspiration from the rapid and efficient movement patterns of the *Acinonyx jubatus*, the aim is to enhance robotic capabilities in terms of speed, stability, and adaptability across varied terrains, thereby bridging the gap between robotic and biological locomotion systems.

## 2. Materials and Methods

### 2.1. Biological Inspiration: Acinonyx jubatus Locomotive Framework

The *Acinonyx jubatus* (cheetah), renowned as the fastest terrestrial quadruped, is capable of reaching peak speeds of 29 m/s during short sprints and covering distances up to 500 m. Remarkably, it can also accelerate from 0 to 100 km/h within just 3 s. An examination of the cheetah’s skeletal muscle anatomy provides insights into its extraordinary locomotive capabilities. One of the key functions of locomotive muscles is to support the animal’s weight by counteracting ground reaction forces and joint torques during stance phases. Another critical requirement for high-speed locomotion is the ability to rapidly swing the limbs and reposition them for the ensuing stride. This agility depends on several factors, including the limbs’ moment of inertia and the muscle moment arms; a smaller moment arm allows for greater changes in joint rotation given a change in muscle length [22].

**Figure 1:**
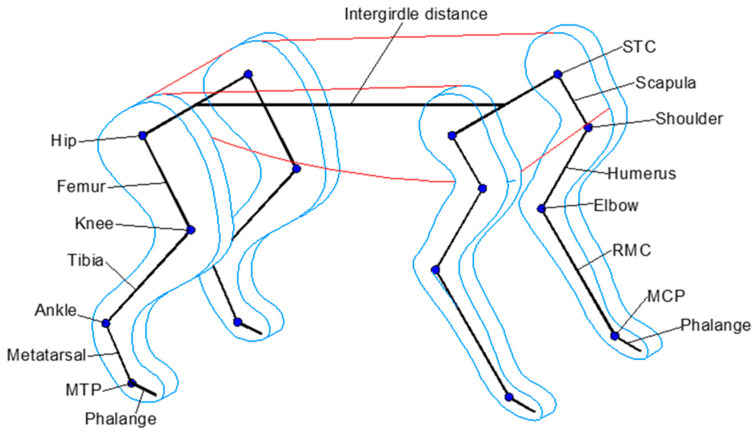
An anatomical illustration of the *Acinonyx jubatus* showcasing the locomotion structure. Key components labeled include the Scapula–Thoracic Cage (STC), scapula, shoulder, humerus, elbow, radius metacarpals (RMCs), Metacarpal–Phalangeal (MCP) joints, hip, femur, knee, tibia, ankle, metatarsal, Metatarsal–Phalangeal (MTP) joints, and the intergirdle distance, modeled in the prototype presented in this paper. However, the segment from the metatarsal to the phalange was considered as a fixed joint.

The cheetah’s forelimb is composed primarily of the scapula, humerus, radius, metacarpals (RMCs), and phalanges, together forming the Scapula–Thoracic Cage (STC), and the shoulder, elbow, and Metacarpal–Phalangeal (MCP) joints (see Figure 1). This limb assembly is supported by approximately 30 muscles, showing no significant reduction in muscle mass from proximal to distal areas. This is because the forelimbs play a crucial role in deceleration, more so than the hindlimbs. Conversely, the cheetah’s hindlimb comprises the femur, tibia, metatarsals, and phalanges, constructing the hip, knee, ankle, and Metatarsal–Phalangeal (MTP) joints (Figure 1). It consists of approximately 37 muscles, exhibiting a notable reduction in muscle mass from proximal to distal areas, with many distal muscles connected by long tendons to minimize the limb’s moment of inertia [23,24]. To make the analysis of the system simpler, each joint was considered to have only one degree of freedom. Furthermore, the body segments and legs were treated as rigid bodies in this model.

Table 3 presents the anatomical measurements of the cheetah’s body structure, including the mean values and standard errors. The intergirdle distance is defined as the length between the uppermost point of the scapula and the hip joints, while the scapula length is measured separately from the other forelimb segments [18]. These parameters and proportions were used as reference to build the *Acinonyx jubatus* prototype presented in this paper on a scale of 1:3.

**Figure 2:**
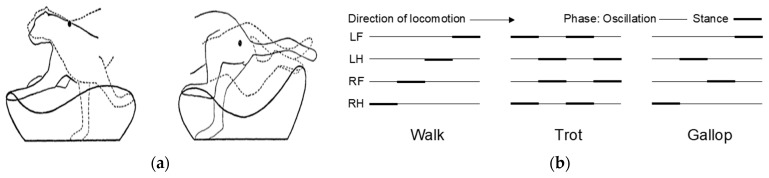
A locomotion analysis of the cheetah’s and mammalian gait patterns: (**a**) depicts the paths followed by the cheetah’s legs in relation to approximate limb pivots at the shoulder and hip, taken from [25]; (**b**) illustrates phase diagrams of typical mammalian locomotion patterns, specifically walking, trotting, and galloping, delineating the transition between stance and oscillation phases for each limb.

The cheetah employs a rotary gallop, where the sequence of footfalls touches the ground in a rotating pattern from the forelimbs around the body to the hindlimbs. The forelimbs make initial contact with the ground when the cheetah transitions from the flight phase to striking the ground. The trajectories of the forelimb and hindlimb are delineated with respect to fixed points located at the shoulder and hip, respectively. As depicted in Figure 2a, the stance phase is when the limb is in contact with the ground, and the oscillation phase is when the limb is airborne and swinging back, tracing a closed curve [25]. Mammals exhibit typical locomotion patterns such as walking, trotting, and galloping, which are distinguished by the phase relationships of limb movements. Figure 2b illustrates the phase diagrams for these common mammalian locomotion patterns. In these diagrams, LF denotes the left forelimb, LH signifies the left hindlimb, RF represents the right forelimb, and RH stands for the right hindlimb. The bold lines indicate the stance phases, while the thinner lines depict the oscillation phases during locomotion.

To generate the trajectories of the limbs and the temporal variation in each joint angle, we modeled each limb as three segments with the foot treated as a rigid, nonarticulated unit. The proportional lengths of these segments were determined using the skeletal measurements of the cheetah as in Table 3. We normalized the entire gait cycle into 19 successive positions, evenly spaced in time, to ensure consistency. Joint angles were measured in a counter-clockwise direction from the horizontal axis of each limb segment at each of these standardized positions.

**Figure 3:**
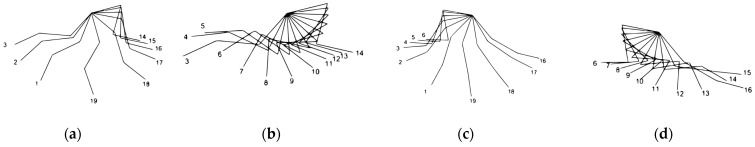
Limb trajectories of a cheetah during different gait cycles, traced from the pivotal scapula point: (**a**,**b**) illustrate the hindlimb trajectory throughout the gait cycle; (**c**,**d**) show the forelimb trajectory during the gait cycle. Each panel captures a phase of movement, highlighting the interplay of limbs during natural locomotion and showcasing the biomechanics in each stride.

Our research draws significantly on the work of Hildebrand [25], who provided the first quantitative descriptions of cheetah locomotion using high-speed film analysis. Hildebrand meticulously documented the limb trajectories and joint movements of cheetahs, identifying characteristic oscillatory patterns in the forelimbs and hindlimbs. These observations have been instrumental in shaping our understanding of the dynamic and highly efficient gait mechanisms in one of the fastest terrestrial animals. To obtain the patterns, the paths followed by the cheetah’s joints relative to the pivotal point, the scapula, were traced. This analysis resulted in 19 dynamic trajectories, as illustrated in Figure 3 adapted from [26]. Each subfigure captures a specific phase of the gait cycle, showing the progressive positions of limbs over a full stride.

**Figure 4:**
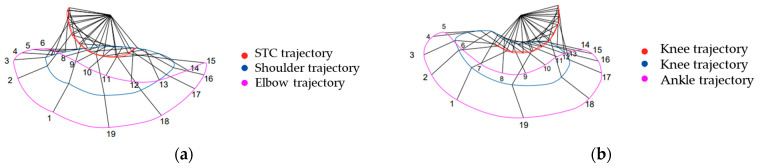
(**a**) Depicts the trajectory of forelimb joints including the STC, shoulder, and elbow throughout the gait cycle. The respective paths are marked with distinct colors: STC trajectory in red, shoulder trajectory in blue, and elbow trajectory in pink. (**b**) Showcases the trajectory of hindlimb joints—the hip, knee, and ankle. Each joint’s path is visually differentiated: hip trajectory in red, knee trajectory in blue, and ankle trajectory in pink, tracing the intricate movement patterns over the course of the gait cycle.

From these trajectories, patterns were derived that outline a cycle formed by the limbs oscillating during the gait cycle. These contours capture the dynamic rhythm of limb gaits throughout different phases of motion, as illustrated in Figure 4 adapted from [26].

As illustrated in Figure 4, the contours can be described and mathematically modeled. To translate these biological insights into our robotic design, we employed CPGs modeled on Wilson–Cowan neural oscillators. The choice was driven by their proven capability in simulating rhythmic and coordinated neural activities that are fundamental to locomotive processes in vertebrates. By adapting these models to robotic applications, we aimed to capture the essence of cheetah locomotion—specifically, the smooth and coordinated movements that define their running gait. In our implementation, the trajectories and joint movements described by Hildebrand were analyzed to determine the key parameters for our CPG model [24,25,26]. We focused on the oscillatory movements of three critical joints—the hip, knee, and ankle. Our analysis revealed that the hip joint predominantly exhibits simple rotational movements, forming arc-like paths that propel the body forward. Conversely, the knee and ankle joints demonstrate complex trajectories that involve compound movements essential for absorbing impact and providing thrust.

Our study combines biomechanical insights and computational models, particularly Wilson–Cowan oscillators, to synthesize cheetah-like gait patterns that closely align with empirical data from Hildebrand’s research. Figure 3a,b illustrate the joint angles and the successive positions of the forelimb and hindlimb segments, respectively, during a high-speed gallop. These figures elucidate the dynamic postures that contribute to the cheetah’s ability to achieve rapid acceleration and high velocity, highlighting the intricate biomechanics involved in one of nature’s most efficient terrestrial locomotion. Notably, at the instances corresponding to positions 18, 19, and 1, the foot engages with and disengages from the ground, signifying the transition between the stance and swing phases of the gait cycle [26].

**Figure 5:**
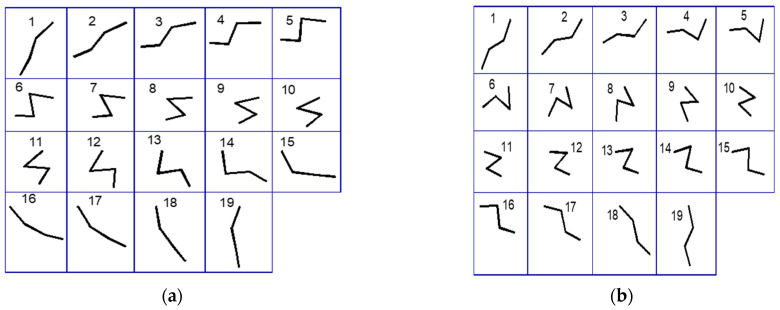
The sequential positioning of limb segments throughout the gait cycle: (**a**) illustrates the positions of the segments for the forelimb during the gait cycle; (**b**) demonstrates the positions of the segments for the hindlimb during the gait cycle.

To implement the kinematic movements of the cheetah gait, the mapped foals were adapted to the inter-limb coordination and phase distribution that a cheetah exhibits during different locomotion states. For instance, when a cheetah transitions from a walk to a gallop, there is a distinctive change in the coordination and timing of its limbs. To establish the authenticity of these gait patterns (natural gait motions), we referenced detailed biomechanical analyses of locomotion based on the studies presented by M. Hildebrand in [25], which provided quantitative data on limb movements and body mechanics during various gaits. The gait patterns shown in Figure 5a,b are thus a reflection of these empirical findings, demonstrating a link between the model output and the cheetah natural gait motions.

**Figure 6:**
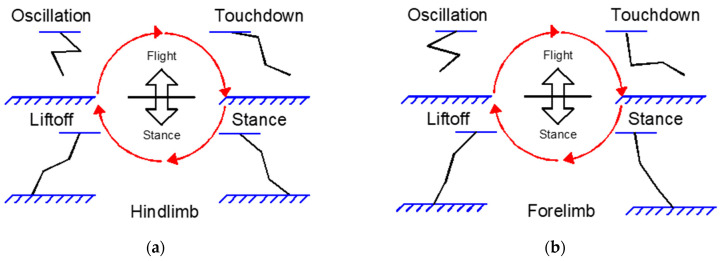
An analysis of the leg movement steps during the gait cycle: (**a**) depicts the flight phase of the gait cycle, highlighting the oscillation step where the leg is positioned to avoid the ground and the touchdown step where a suitable angle for landing is selected; (**b**) shows the stance phase of the gait cycle, emphasizing the stance step involving weight support and the liftoff step preparing for the transition back to the flight phase.

To simplify, leg movements were categorized into four steps: oscillation, touchdown, stance, and liftoff. The oscillation and touchdown steps occur during the flight phase of the gait, where the primary objective is to clear the ground and position the leg at an optimal angle for the impending touchdown. Conversely, the stance and liftoff steps happen during the stance phase, which involves supporting the body’s weight and preparing for the next cycle of movement. This segmentation of the gait cycle is depicted in Figure 6. The delineation of these steps allows for a focused analysis of each critical phase, providing a structured approach to study the biomechanics involved in the gait cycle and its impact on the efficiency of locomotion [3].

Before the forelimb contacts the ground, it starts to retract—a behavior frequently observed in quadrupedal locomotion [27]. This pre-landing retraction slows down the foot’s velocity relative to the ground, effectively mitigating the impact at the point of touchdown and minimizing potential injury or discomfort from the force of the landing [28]. Moreover, the retraction of the leg prior to ground contact enhances the running stability of the animal, allowing for more precise and controlled movements [29]. In contrast, the hindlimbs serve a different, yet equally critical, role. Their primary function is to deliver powerful thrusts to the body, generating substantial acceleration. This propulsive force is essential for initiating and maintaining high-speed pursuits, enabling the animal to rapidly increase its velocity and maneuver with agility. Understanding the distinct roles of the fore- and hindlimbs in movement not only provides insight into the mechanics of quadrupedal motion but also informs the design and control of robotic systems aiming to emulate these natural dynamics.

As we conclude our study of the biomechanical aspects and their implications on stability and propulsion in quadruped locomotion, the following section will explore the mechanisms underlying these intricate motions, presenting the CPGs that are the fundamental components of rhythmic motor pattern production in both biological and robotic subjects.

### 2.2. Mechanism Modeling for Autonomous Locomotion Control Based on Wilson–Cowan Neural Oscillators

CPGs are specialized neural circuits that, once activated, are capable of generating rhythmic motor patterns for activities such as walking, breathing, flying, and swimming, independent of sensory inputs that provide specific timing information [30]. The CPG model, inspired by the Wilson–Cowan neural oscillator, is composed of excitatory and inhibitory neurons interconnected through synaptic links [31]. This configuration and functionality in producing autonomous locomotion patterns are described by the following differential Equations (1)–(3):

(1) $$T_{u}\frac{du}{dt}=-u+f_{µ}(au-bv+S_{u})$$

(2) $$T_{v}\frac{dv}{dt}=-v+f_{µ}(cu-dv+S_{v})$$

(3) $$f_{µ}(x)=tanh(µx)$$

where *u* and *v* represent the activities of groups of excitatory and inhibitory neurons, respectively. The parameters *a* through *d* characterize the coupling strength between these neuronal groups, while *b* denotes the coupling strength from *v* to *u* and *c* from *u* to *v*. *S_u_* and *S_v_* are external inputs, and *T_u_* and *T_v_* are the respective time constants. The transfer function *fµ*(*x*) is defined by the hyperbolic tangent tanh(*µx*), where *µ* is the control parameter. Depending on these parameters, the Wilson–Cowan neural oscillator can exhibit a variety of behaviors [31].

In the context of a Wilson–Cowan neural oscillator controller for a quadruped robot, the output of the neural oscillator is the difference between the activities of the excitatory neuron and the inhibitory neuron, denoted as *y_out_* = *u* − *v*. In the CPG controller we propose, each Wilson–Cowan neural oscillator is tasked with controlling an individual limb. The movement of the limbs is orchestrated by four Wilson–Cowan neural oscillators, with the output from each oscillator used to regulate the synchronized motion of its corresponding limb. The connection weight matrix *w_ij_* is used to generate rhythmic movement patterns. The dynamics of the model are captured by Equations (4) and (5) [20]:

(4) $$T_{u_{i}}\frac{du_{i}}{dt}=-u_{i}+f_{µ}(au_{i}-bv_{i}+\sum_{j=1}^{n}w_{ij}u_{j}+S_{u_{i}})$$

(5) $$T_{v_{i}}\frac{dv_{i}}{dt}=-v_{i}+f_{µ}(cu_{i}-dv_{i}+\sum_{j=1}^{n}w_{ij}v_{j}+S_{v_{i}})$$

where *i* and *j* denote the distinct oscillators within the system, with *w_ij_* representing the connection weight between these oscillators $W\in R^{4\times 4}$, and $y_{out}^{i}=u_{i}-v_{i}$ with *i*, *j* = 1, 2, 3, 4. Using four interconnected Wilson–Cowan neural oscillators, we construct a fully symmetrical CPG network designed for a quadruped robot. This network symmetry ensures that the generated locomotive patterns are harmonized across all four limbs, thereby stabling, and coordinated movement akin to biological quadrupeds.

In our approach, the connection weight matrix rows represent the four states of two neurons within a single oscillator, and the columns correspond to the four limbs of the robot. Our quadruped robot is designed to emulate four typical movement patterns, commonly known as gaits, which include walking, trotting, and galloping. A gait is characterized by various parameters such as the order of limb phases, duty factor, cycle time, and velocity. In the movement of quadruped robots, the phase order is particularly crucial in defining the specific pattern of each gait. We represent the weight connection matrices for the gaits of walking, trotting, pacing, and galloping in Equations (6)–(9).

The weight matrices *W_walk_*, *W_trot_*, *W_pace_*, *W_gallop_* are input parameters of the CPG model, directing the phase and coordination of limb movements to emulate the distinct gaits. These weight matrices were formulated through an iterative process that incorporates empirical biomechanical data with the theoretical modeling of animal locomotion, presented by Li, B., Li, Y., and Rong, X in [20]. For each gait, the matrix is constructed to initiate the CPG network to produce the desired phase relationships and timing patterns that align with those observed in the cheetah’s natural movement. For instance, the matrix *W_walk_* induces a limb coordination pattern that reflects the staggered, yet stable, gait of walking, whereas *W_gallop_* synchronizes limb movements to recreate the powerful, synchronized motions of a galloping cheetah. Based on empirical observations of cheetah locomotion and existing biomechanical models in the literature, matrices of weights that determine CPG results were computed to replicate gait coordination. The artificial gait patterns generated by these matrices are not mere approximations but are the result of the adaptability of biological gait patterns.

For the walking gait, represented by *W_walk_* in Equation (6), the zero elements indicate no direct coupling between diagonal limb pairs, allowing for the alternate movement typical in a walking gait. This is characterized by a sequence where only one foot is lifted off the ground at any given time, ensuring stability. The trotting gait, defined by *W_trot_* in Equation (7), involves simultaneous movements of diagonal limb pairs. The matrix depicts off-diagonal negative elements that suggest an inhibitory coupling between diagonal limbs, leading to their alternate action and the characteristic two-beat pattern. The pace gait, described by *W_pace_*, is characterized by the synchronized movement of the limbs on the same side of the body. This is reflected in the matrix by the positive off-diagonal elements between the left forelimb and left hindlimb, and the right forelimb and right hindlimb, enabling the lateral swinging movement central to the pace gait. Lastly, the gallop gait, presented by *W_gallop_*, necessitates a more complex synchronization, with the forelimbs and hindlimbs operating in two distinct pairs. The positive elements between the forelimb pairs and hindlimb pairs foster this synchronous activity within each pair, resulting in the explosive and dynamic movement that defines a gallop. These matrices were not arbitrarily designed; rather, they were grounded in the foundational work from the dynamics in Wilson–Cowan neural oscillators [32] and Li, B., Li, Y., and Rong, X. [20], based on the movement patterns related to the spine and shoulders. According to this study, movement patterns during specific periods describe the four types of gaits, which are then correlated with the CPG patterns.

(6) $$W_{walk}=\left[\begin{array}{cc} 0 & \begin{array}{ccc} -0.1 & -0.1 & -0.1 \end{array}\\ \begin{array}{c} -0.1\\ -0.1\\ -0.1 \end{array} & \begin{array}{ccc} \begin{array}{c} 0\\ -0.1\\ -0.1 \end{array} & \begin{array}{c} -0.1\\ 0\\ -0.1 \end{array} & \begin{array}{c} -0.1\\ -0.1\\ 0 \end{array} \end{array} \end{array}\right]$$

(7) $$W_{trot}=\left[\begin{array}{cc} 0 & \begin{array}{ccc} -0.1 & 0.1 & -0.1 \end{array}\\ \begin{array}{c} -0.1\\ 0.1\\ -0.1 \end{array} & \begin{array}{ccc} \begin{array}{c} 0\\ -0.1\\ 0.1 \end{array} & \begin{array}{c} -0.1\\ 0\\ -0.1 \end{array} & \begin{array}{c} 0.1\\ -0.1\\ 0 \end{array} \end{array} \end{array}\right]$$

(8) $$W_{pace}=\left[\begin{array}{cc} 0 & \begin{array}{ccc} -0.1 & -0.1 & 0.1 \end{array}\\ \begin{array}{c} -0.1\\ -0.1\\ 0.1 \end{array} & \begin{array}{ccc} \begin{array}{c} 0\\ 0.1\\ -0.1 \end{array} & \begin{array}{c} 0.1\\ 0\\ -0.1 \end{array} & \begin{array}{c} -0.1\\ -0.1\\ 0 \end{array} \end{array} \end{array}\right]$$

(9) $$W_{gallop}=\left[\begin{array}{cc} 0 & \begin{array}{ccc} 0.1 & -0.1 & -0.1 \end{array}\\ \begin{array}{c} 0.1\\ -0.1\\ -0.1 \end{array} & \begin{array}{ccc} \begin{array}{c} 0\\ -0.1\\ -0.1 \end{array} & \begin{array}{c} -0.1\\ 0\\ 0.1 \end{array} & \begin{array}{c} -0.1\\ 0.1\\ 0 \end{array} \end{array} \end{array}\right]$$

However, variations in the output gain can lead to differences in the amplitudes before and after transitions between gaits. To equalize the amplitude of the gaits, we have introduced a normalization process for the outputs of the CPG model. This normalization ensures consistent amplitude levels across different gaits, assisting in smoother transitions and more uniform locomotion patterns in the quadruped robot to process the data. The CPG output *y_out_* is normalized through Equation (10).

(10) $$y_{out}\left(i\right)=a+\frac{(y_{out}\left(i\right)-\min\left(y_{out}\left(i\right)\right))}{\max\left(y_{out}\left(i\right)\right)-\min\left(y_{out}\left(i\right)\right)}(b-a)$$

where a is the minimum value of the normalized outputs, while b is defined as the maximum. Therefore, a=−1,b=1, and output is normalized to the interval [−1,+1]. In the following section, we delve into the forward kinematics of the quadruped robot, focusing on a simplified model and the formulation of homogeneous transformation matrices (HTMs). This part of the paper provides an in-depth analysis of the robot’s forelimbs, hindlimbs, and body, offering a detailed perspective on its mechanical structure and movement capabilities.

### 2.3. Kinematic Analysis of Quadruped Locomotion

To control the quadruped effectively, it is essential to understand the relationships between the movements of the joints (inputs) and the motions of the end effector (outputs), as the former directly governs the latter. Consequently, the study of kinematics is critical when transformations must be performed between the coordinate reference systems associated with the various links of the robot. A robot consists of multiple links connected serially through joints. The degrees of freedom (DOFs) of the robot depend on the number and types of these links and joints, as well as the kinematic chain of the robot. This kinematic framework forms the basis for analyzing and simulating the mechanical movements required for the robot to perform tasks accurately [21,28]. In a three-dimensional Cartesian space, a rigid body possesses six DOFs. This implies that the position of the body can be described using three translational coordinates and its orientation through three rotational coordinates. From a kinematic perspective, two or more components are considered as a single link when they are connected without any relative movement between them. The links of a robot are joined through pairs or kinematic joints, which provide the physical constraints necessary for the relative motion between the links. The form of the contact surface—which can be a surface, a line, or a point—between the joints determines the kind of relative motion. A rotary pair, hinge, or pin joint allows two paired links to rotate relative to each other around the axis of the joint. This rotation is a critical aspect of robotic articulation, enabling the quadruped to achieve complex movements and orientations. Also, another aspect to consider is the kinematic chain. A kinematic chain refers to a series of links interconnected by joints. In cases where each link in a kinematic chain is connected to a maximum of two other links, the chain is classified as a simple kinematic chain [4].

The movement of a rigid body can be described using six coordinates, involving both translational and rotational movements. These coordinates define the dynamics of the manipulator (robotic arm), tools (end effectors), and workpieces (items or materials being processed), which are essential components in robotic operations. Subsequently, a Cartesian coordinate system is fixed to the moving body (the quadruped), enabling a description of its posture and orientation. This dual-system approach allows for the tracking and control of the prototype orientation and position in space, facilitating manipulations and interactions within the quadruped system. The position of any point *P* on a moving rigid body relative to a fixed reference system can be described using a three-dimensional Cartesian vector ***p***, if the coordinates of point P or the components of vector *p* in a fixed system F are as follows: ***p***_*x*_, ***p***_*y*_, and ***p***_*z*_, denoted by Equation (11).

(11) $${\left[p\right]}_{F}=\left[\begin{array}{c} p_{x}\\ p_{y}\\ p_{z} \end{array}\right]$$

where the subscript *F* refers to the reference system in which the vector ***p*** is represented. The subscripts *x*, *y*, and *z* denote the projections of the position vector ***p*** onto the coordinate axes of the fixed reference system, namely along the *X*-, *Y*-, and *Z*-axes, respectively. Therefore, the vector ***p*** can be expressed as Equation (12).

(12) $$p=p_{x}x+p_{y}y+p_{z}z$$

where *x*, *y*, and *z* denote the unit vectors along the *X*-, *Y*-, and *Z*-axes of system *F*, respectively. Their representations in system *F* are presented in Equation (13).

(13) $${\left[x\right]}_{F}=\left[\begin{array}{c} 1\\ 0\\ 0 \end{array}\right],\;{\left[y\right]}_{F}=\left[\begin{array}{c} 0\\ 1\\ 0 \end{array}\right],\;{\left[z\right]}_{F}=\left[\begin{array}{c} 0\\ 0\\ 1 \end{array}\right]$$

The subsequent parameters to be defined are the rotations of the rigid body, which are essential for establishing the Denavit–Hartenberg (DH) parameters. Assuming that a reference system *M* initially coincides with the fixed reference system *F*, the system *M* is then rotated by an angle *α* around the *Z*-axis. The unit vectors of the oriented system *M* can be described in terms of their components in the system *F* as in Equation (14).

(14) $${\left[u\right]}_{F}=\left[\begin{array}{c} C_{\alpha }\\ S_{\alpha }\\ 0 \end{array}\right],\;{\left[v\right]}_{F}=\left[\begin{array}{c} -S_{\alpha }\\ C_{\alpha }\\ 0 \end{array}\right],\;{\left[w\right]}_{F}=\left[\begin{array}{c} 0\\ 0\\ 1 \end{array}\right]$$

where *S* ≡ sine, and *C* ≡ cosine. Therefore, the rotation matrix, denoted as **Q**Z, is calculated as follows in Equation (15).

(15) $$Q_{z}=\left[\begin{array}{ccc} C_{\alpha } & -S_{\alpha } & 0\\ S_{\alpha } & C_{\alpha } & 0\\ 0 & 0 & 1 \end{array}\right]$$

Once the translation and rotation parameters are defined, the coordinate transformations necessary for analyzing the motion between any two systems in robotic kinematics are succinctly encapsulated through the homogeneous transformation matrix (HTM). This matrix is represented as in Equation (16).

(16) $$[p_{F}^{1}]=\left[\begin{array}{cc} Q & o_{F}^{0}\\ 0 & 1 \end{array}\right]\left[\begin{array}{c} {\left[p^{\prime }\right]}_{M}^{1}\\ 1 \end{array}\right]$$

where ***o*** ≡ [0 0 0]**^T^** is the zero vector, which streamlines the integration of both translational and rotational components into a single matrix operation, eliminating the need for separate operations. Here, the 4 × 4 matrix, **T**, enables the transformation of vectors from the moving system *M* to the fixed system *F* through simple matrix multiplication.

This approach is particularly effective in the control of robotic quadrupeds, which typically consist of several links connected via single-degree-of-freedom joints. To precisely control the end effector relative to the base, it is crucial to describe the coordinate transformations for each link. The quadruped prototype is composed of *n* + 1 links, interconnected by *n* = 3 joints. The DH convention not only simplifies the description and computation of the kinematic chain but also enhances the accuracy and efficiency in controlling the robot’s configuration and motion.

**Figure 7:**
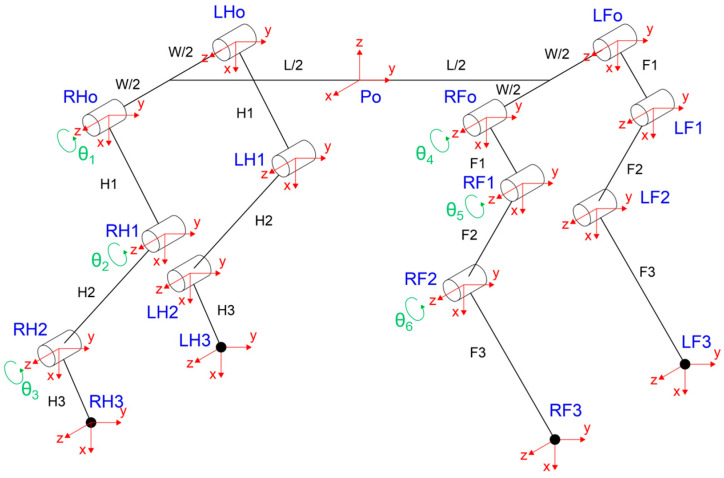
A simplified kinematic model of a quadruped robot, illustrating the principal axis system at the body center (Po), the segmentation of the limbs, and the degrees of freedom at each joint. The model highlights the structural symmetry and articulation points across the robot’s body, with ‘L’ and ‘W’ denoting the robot’s length and width, respectively.

To describe the forward kinematics of the robot, the forelimbs, hindlimbs, and body were analyzed. This analysis led to the development of a simplified model and the derivation of HTMs, which are used to determine the position and orientation of the effectors at the end of each limb, relative to the robot center. Figure 7 presents the reduced model with the anatomical arrangement and articulation points. The body center, denoted as Po, is the origin of the principal axis system. From Po, the model extends symmetrically, with lengths L/2 toward both the anterior and posterior parts and widths W/2 towards the left and right sides. The joints and limbs are marked, with the limbs segmented into three parts: proximal (H1, F1), medial (H2, F2), and distal (H3, F3) sections. The joints between these segments are indicated by green circles with angles θ_1_ to θ_6_, representing the degrees of freedom for rotational movement. Connection points on the body for the limbs are denoted as LHo, RHo, LFo, and RFo. Three degrees of freedom (DOFs) are assigned to each limb in the model, for a total of 12 DOFs.

Three rotating joints—RFo, RF1, and RF2—represent the Scapula–Thoracic Cage (STC), the shoulder, and the elbow in the simplified model’s forelimb. RF3 indicates the location of the effector. The corresponding segments for these joints are labeled F1, F2, and F3, which stand for the scapula, humerus, and radius metacarpals (RMCs), respectively. Each joint is capable of angular movement denoted by θ_4_, θ_5_, and θ_6_ around the *Z*-axis of the coordinate system. Similarly, the hindlimb model is constructed with three rotational joints that correspond to the hip, knee, and ankle, labeled RHo, RH1, and RH2, with RH3 marking the effector. The associated limb segments are identified as H1, H2, and H3, which refer to the femur, tibia, and metatarsals, respectively. These joints exhibit angular displacements denoted by θ_1_, θ_2_, and θ_3_, each rotating around the *Z*-axis of their respective axis systems. Lastly, the HTMs from the center of the robot to each one of the effectors at the end of each limb are as follows: for hindlimb RH and LH, with C_12_ ≡ Cos (θ_1_ + θ_2_), C_123_ ≡ Cos (θ_1_ + θ_2_ + θ_3_), S_12_ ≡ Sin (θ_1_ + θ_2_), S_123_ ≡ Sin (θ_1_ + θ_2_ + θ_3_). Equations (17) and (18) present the RH_3_ and LH_3_ matrix.

(17) TRH3Po=00S123C1231W/20H3S123+H2S12+H1S1−L/2−C123S12300 0−H3C123−H2C12−H1C101

(18) TLH3Po=00S123C1231−W/20H3S123+H2S12+H1S1−L/2−C123S123000−H3C123−H2C12−H1C101

For forelimb RF and LF, presented in Equations (19) and (20), with C_45_ ≡ Cos (θ_4_ + θ_5_), C_456_ ≡ Cos (θ_4_ + θ_5_ + θ_6_), S_45_ ≡ Sin (θ_4_ + θ_5_), S_456_ ≡ Sin (θ_4_ + θ_5_ + θ_6_)

(19) TRF3Po=00S456C4561W/20F3S456+F2S45+F1S4+L/2−C456S456000−F3C456−F2C45−F1C401

(20) TLF3Po=00S456C4561−W/20F3S456+F2S45+F1S4+L/2−C456S456000−F3C456−F2C45−F1C401

## 3. Results

**Figure 8:**
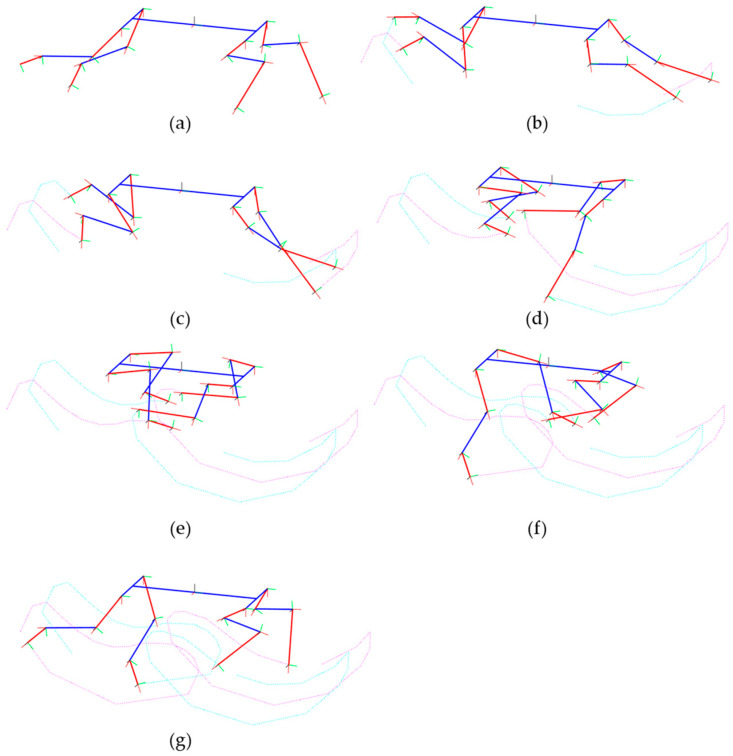
Sequential kinematic simulations of the quadruped robot during a gallop gait, illustrating the dynamic posture and limb positioning at various instants: (**a**) 1/19, (**b**) 4/19, (**c**) 7/19, (**d**) 10/19, (**e**) 13/19, (**f**) 16/19, and (**g**) 19/19. Each image captures the complexity and synchronization required for the robot to replicate the gait effectively. Red lines denote left limb trajectories, while green trajectories denote right limb movements.

The quadruped robot’s walk, trot, and gallop were visualized by kinematics simulation using the previously established HTM. The simulation, depicted in Figure 6, graphically represents the robot’s limbs and body using lines in blue and red to differentiate each segment. Additionally, coordinate systems are assigned to each joint and effector, allowing for an accurate depiction of their position and orientation. In Figure 8, the simulation illustrates the quadruped robot during a gallop gait at selected intervals of the gait cycle. The figure highlights the paths followed by the limb effectors and the dynamic alignment of the segments.

**Figure 9:**
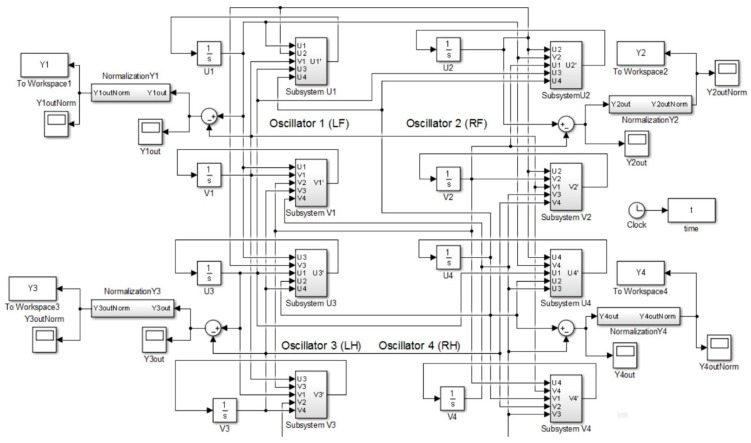
A block diagram of the CPG model, outlining the neural oscillators for each limb and their interconnections, and it demonstrates the flow from differential equations to the normalized output for each oscillator.

**Figure 10:**
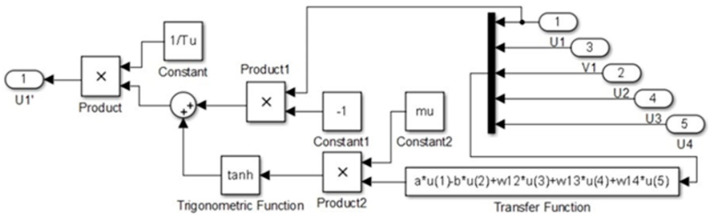
U*i* subsystem.

**Figure 11:**
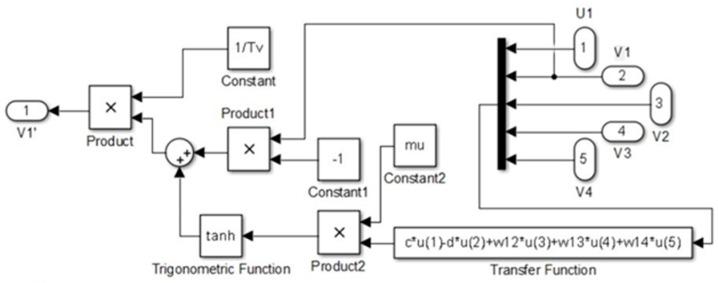
V*i* system.

**Figure 12:**
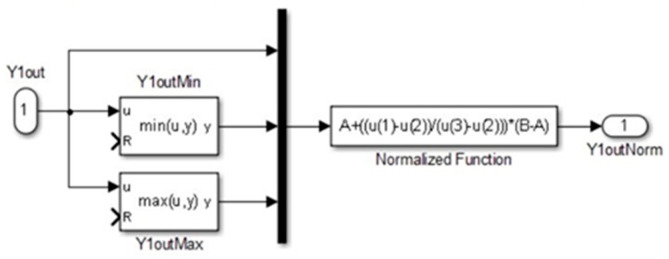
Normalization system.

The simulation of the CPG controller is depicted in Figure 7, including four neural oscillators, one for each limb: left forelimb (LF), right forelimb (RF), left hindlimb (LH), and right hindlimb (RH). These oscillators are tasked with solving the differential equations for the neuron activities U*_i_* and V*_i_*, with *i* ranging from 1 to 4. The simulation illustrates how the oscillators are interconnected. The output Y*_i_* of each oscillator is calculated as the difference between the activities U*_i_* and V*_i_*, and this output is subsequently normalized Y*_iNorm_* to a predefined interval. The models presented in Figure 9, Figure 10, Figure 11 and Figure 12 were modeled in MATLAB Simulink 2021-b. This software platform facilitated the creation of dynamic system models, enabling the simulation and analysis of the complex interactions of the control algorithms that govern the robot’s behavior.

The U*i* neuron subsystems are depicted in Figure 8. This subsystem evaluates the transfer function outlined in Equations (4) and (5), incorporating the normalization parameter *μ* and synaptic weights W*ij*, along with constants a, b, Tu, and Su.

Conversely, in the V*i* neuron subsystems, presented in Figure 9, the inputs include signals V*i* and U*i*. The same transfer function is applied, also using *μ* and W*ij*, but with different constants c, d, Tv, and Sv.

For normalizing the output signal Y*i* (derived from the difference U*i*–V*i*, as illustrated in Figure 10), the process involves determining the signal maximum and minimum values. The normalization function, as specified in Equation (10), is then applied to scale the output within a predefined range [A, B]. The differential equations central to the CPG model were resolved using the fourth-order Runge–Kutta integration method with a simulation step of 0.001 s. The simulation was set to a duration of 10 s with the following parameter settings: Tu = Tv = 0.2, a = 5.5, b = 5.5, c = 2.5 d = 0, Su = Sv = 0, and *μ* = 0.5. By applying weight matrices W*ij* for each gait as outlined in Equations (6)–(9), the model successfully generated the walking, trotting, pacing, and galloping gaits at the onset of the simulation.

**Figure 13:**
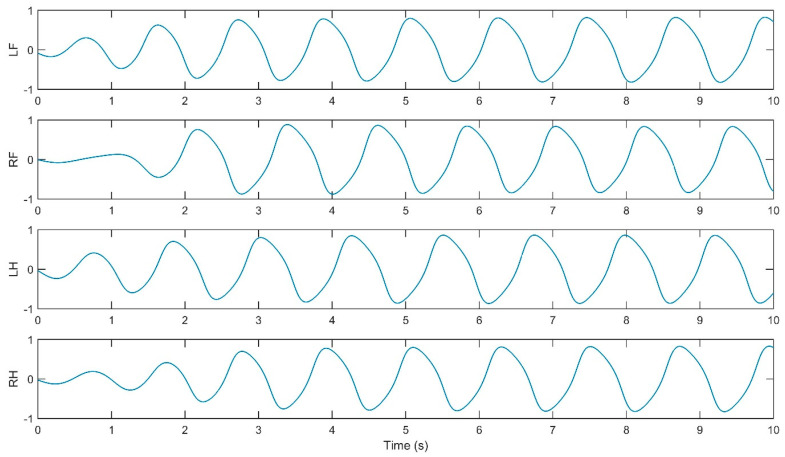
Relative phase CPG signals for walk gait.

**Figure 14:**
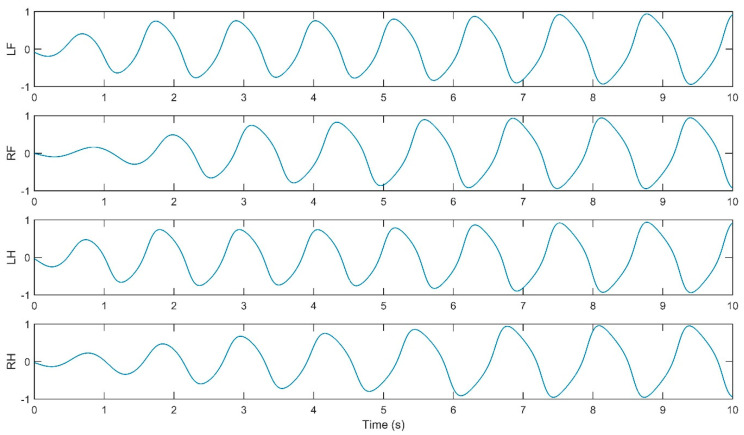
Relative phase CPG signals for trot gait.

**Figure 15:**
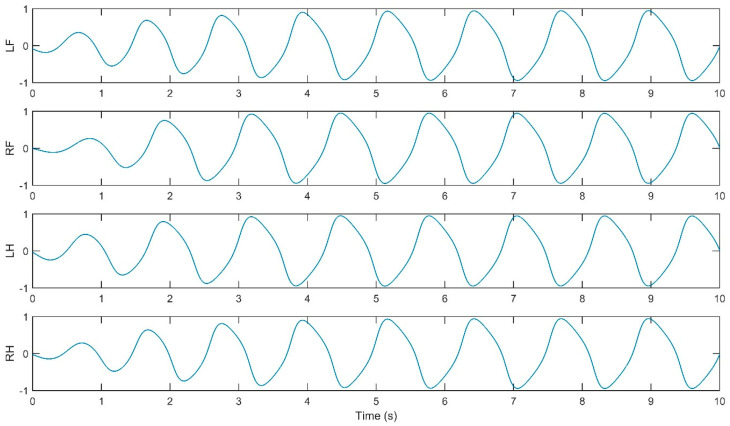
Relative phase CPG signals for pace gait.

**Figure 16:**
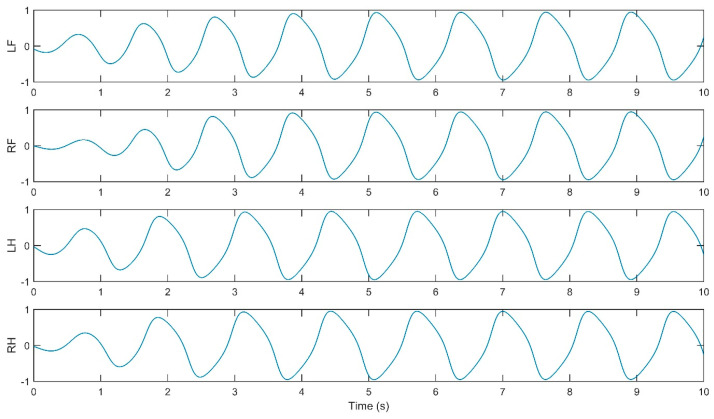
Relative phase CPG signals for gallop gait.

The simulation yielded oscillatory signals from the CPG for each of the quadruped’s legs (i.e., LF, RF, LH, RH), depicted in Figure 11, Figure 12, Figure 13 and Figure 14. Figure 11 shows the walk gait signals, where an offset of a quarter cycle exists between the RF and LH signals and a half-cycle offset between the LH and LF signals (left side legs), as well as between the RF and RH signals (right side legs). Figure 12 presents the trot gait for LH and LF signals closely aligned in phase, like the RF and RH signals, with a quarter-cycle offset between these two pairs. Figure 13 presents the pace gait for when the LF and RH signals are synchronized, as are the RF and LH signals, with a half-cycle offset between these pairs. Lastly, Figure 15 depicts the gallop gait, where the LF and RF signals (forelimbs) are in phase, mirroring the phase alignment of the LH and RH signals (hindlimbs), with a half-cycle offset between these two pairs of signals. Figure 13, Figure 14, Figure 15 and Figure 16 depict the cyclic activity of the CPG for the pace gait. The rhythmic oscillations showcase a synchronized pattern between the limbs on each side for lateral gait movements. Each limb’s CPG signal is in phase with its lateral counterpart while being anti-phase with the diagonally opposite limb, resulting in a movement that mirrors the natural pacing motion found in certain quadrupedal animals. The CPG model supports these rhythmic patterns on the principles of neural oscillations described by Wilson–Cowan. The parameters within the weight matrix *W_walk_*, *W_trot_*, *W_pace_*, *W_gallop_* were derived to ensure the output signals from the CPG would align with the observations of the biological rhythms, presented by Li, B., Li, Y., and Rong, X. [20]. The quadruped limbs, thus, exhibit a pace gait that closely resembles the cheetah’s locomotion, displaying the smooth side-to-side movements essential for high-speed maneuvering.

**Figure 17:**
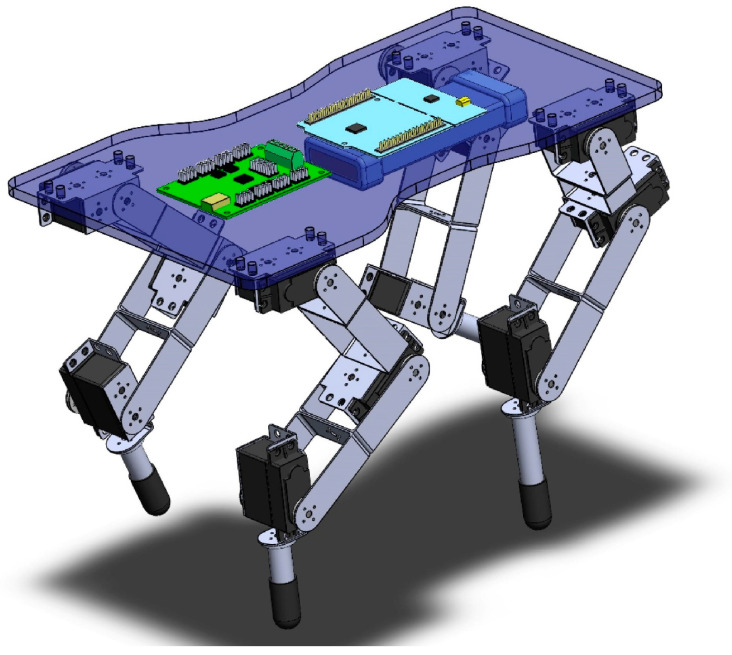
A CAD rendering of the quadruped robot done in SolidWorks 2018, showcasing the chassis, limb assembly with servomotors at each joint, supporting brackets, segmented links representing the limbs, and cylindrical tubes with rubber covers as effectors.

The mechanical design of the robot was modeled at a 1:3 scale of a real cheetah. This scale was selected to balance the proportional accuracy essential for simulating the cheetah’s movement with the practical feasibility of construction and component integration. This scale aids in the replication of biomechanical functions, including speed and agility, within the confines of technical and material limitations. The success of this scaling strategy was further validated by simulation and testing results, which demonstrate the robot’s capability to emulate the cheetah’s distinctive locomotion patterns. Table 4 provides the measured values of the robot segments. The design of the robot features a chassis that is wider at the back than at the front to prevent limb collision. The prototype was equipped with 12 servomotors. Brackets were used to support the servomotors and to form the joints, while links represent the segments of the robot. Additionally, cylindrical tubes with a rubber covering were used as effectors, as presented in Figure 17.

**Figure 18:**
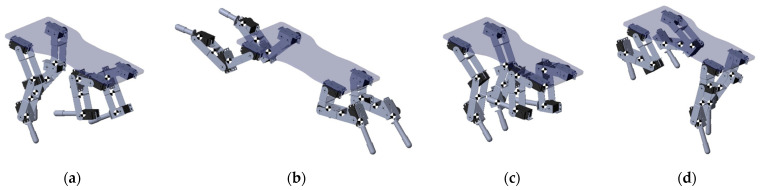
The mechanical simulation of the prototype, (**a**) stance, where the limb is in contact with the ground supporting the robot’s weight; (**b**) liftoff, depicting the initiation of the limb’s ascent from the ground; (**c**) oscillation, showing the limb in mid-air as it prepares for the next step; and (**d**) touchdown, where the limb re-establishes contact with the ground, completing the cycle.

The simulation of the prototype movement across various gaits was carried out in a virtual environment. Physical material properties were considered, such as mass, center of mass, and moment of inertia. Figure 18 presents the movement simulation throughout the gait cycle, highlighting the centers of mass for each limb segment. During the simulation, all parts of the robot were treated as rigid bodies with the chassis grounded at the origin, and factors such as frictional interactions with the ground were not considered for simulation simplification.

**Figure 19:**
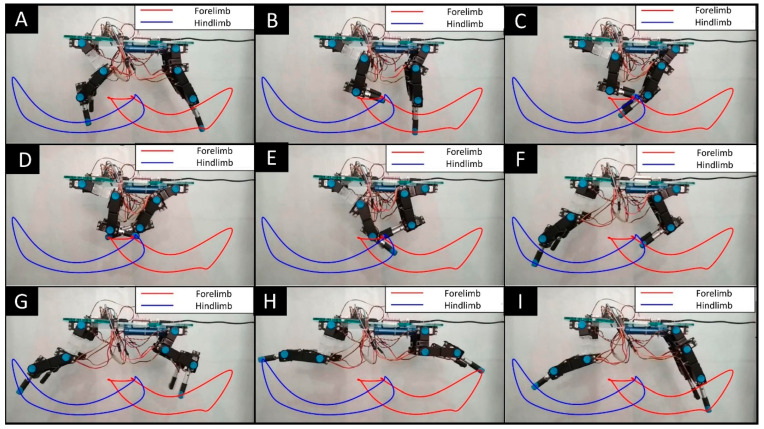
Trajectories of the forelimb (in red) and hindlimb (in blue), as captured in a video at distinct instances within the gait cycle (Frames (**A**)-1, (**B**)-2, (**C**)-3, (**D**)-5, (**E**)-6, (**F**)-8, (**G**)-9, (**H**)-10, (**I**)-11).

To determine the angular positions and trajectories of the limbs during the gait cycle, a video capturing the robot’s movement was recorded, using blue markers placed on the joints and effectors of the limbs. Eleven key frames were extracted from the video at various time intervals. Using image processing techniques, the angles formed with the horizontal plane of the chassis were measured for each frame, allowing for the determination of the angular positions of the STC, shoulder, and elbow joints for the forelimb, as well as the hip, knee, and ankle joints for the hindlimb. Similarly, the trajectories described by the effectors were traced for the forelimb (indicated by a red line) and the hindlimb (indicated by a blue line). These trajectory curves were plotted using spline interpolation to create smooth and continuous paths. The limb trajectories form elongated, semi-circular closed curves with a small intersecting region in the center, as illustrated in Figure 19. This detailed analysis provides valuable insights into the mechanical performance and movement efficiency of the robot’s limbs during locomotion.

**Figure 20:**
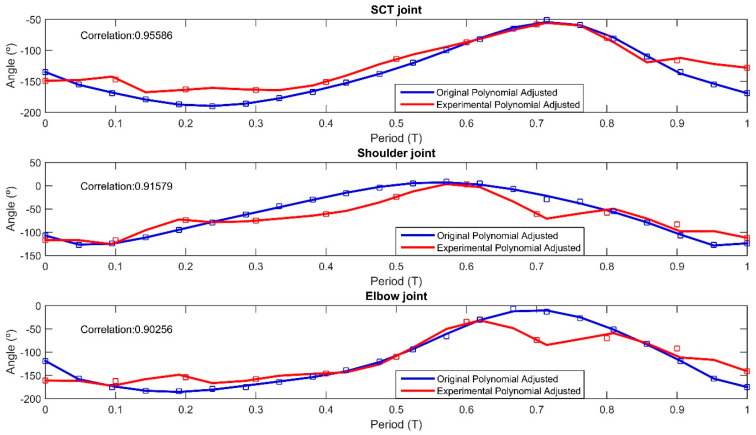
Real (blue line) and experimental angular positions (red line) for forelimb joints in motion cycle.

Comparative graphs were created to compare the theoretical and experimental angular positions of the forelimb and hindlimb joints. Figure 20 presents the angular positions for the STC, shoulder, and elbow joints of the forelimb throughout the gait cycle, with the theoretical data displayed in blue and the experimental data in red. The theoretical graph was plotted using 22 angular positions, while the experimental graph utilized 11 positions. For both datasets, a 10th-degree polynomial was fitted, yielding correlation values of 0.9558 for the STC joint, 0.9157 for the shoulder joint, and 0.9025 for the elbow joint. Figure 19 presents the angular positions of the hip, knee, and ankle joints for the hindlimb during the gait cycle. The correlation results were 0.9560 for the hip joint, 0.7071 for the knee joint, and 0.9460 for the ankle joint, indicating the degree of alignment between the experimental observations and the theoretical model.

**Figure 21:**
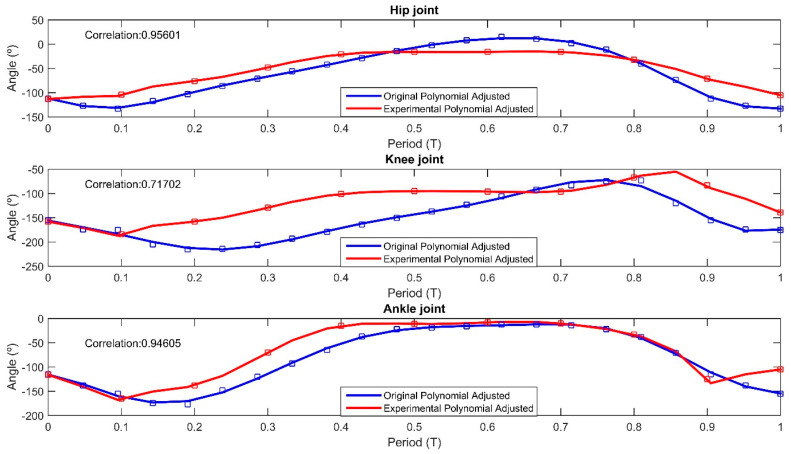
Real (blue line) and experimental angular positions (red line) for hindlimb joints in motion cycle.

Figure 20 and Figure 21 address the comparison between the quadruped movements and the theoretical model, correlating between the computed angles and those captured by computer vision, depicted as contours in red and blue in Figure 19. This visual alignment underscores the accuracy of our prototype to biomimetic principles that guided its design. The contours in Figure 19 represent trajectories, while the prototype’s movements reflect these paths confirming the emulation of natural locomotion. To provide a clear, quantitative perspective, we compared theoretical and computer vision-derived empirical data, showcasing the high correlation in Figure 20 and Figure 21.

**Figure 22:**
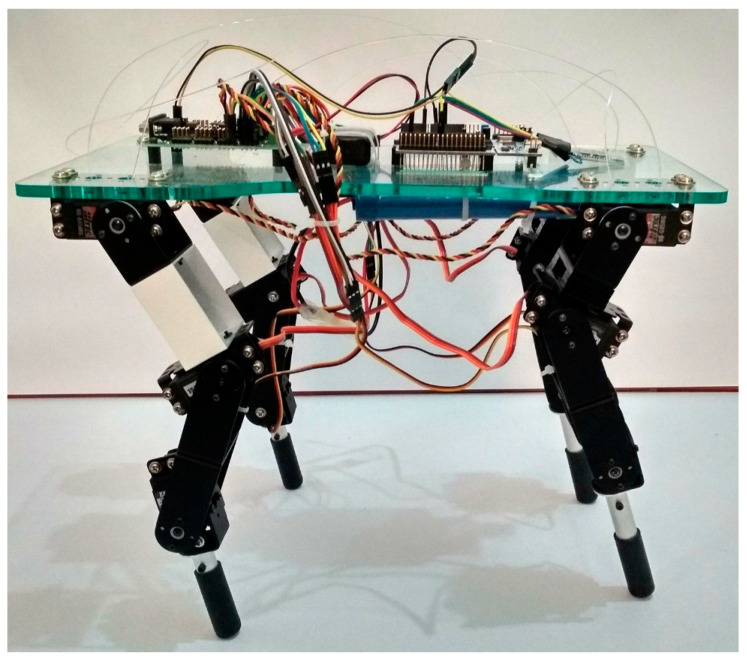
*Acinonyx jubatus* prototype.

In the development of the *Acinonyx jubatus* prototype, attention was given to the selection of materials and components to achieve a design that was not only robust but also lightweight, factors which are critical in replicating the agility and efficiency of the quadruped movement. This is evident in the results depicted in Figure 18 and Figure 19, where the kinematic performance of the robot aligns closely with the modeled predictions. The use of 12 Power HD-1501MG servomotors, characterized by their 17 kg-cm (approximately 235.4 oz-in) torque and 0.14 s per 60 degrees of motion response, served as the actuators. These motors enable the prototype to execute movements with the necessary force and precision while maintaining a swift and fluid motion. The integration of two mainboards, an SSC32 for servomotor control and a Nucleo STM32 for algorithm implementation, forms the control system. These boards, mounted atop the chassis, work in unison to process and execute the control algorithms that drive the robotic limbs through their naturalistic gait cycles. The power efficiency is further optimized by the 7.2 V LiPo battery, which supplies energy without contributing excessive weight, ensuring the robot’s movements remain dynamic and energy-efficient. The completed prototype, depicted in Figure 22, measures 35.5 cm in length, 21 cm in width at the rear, 17 cm in width at the front, and stands 27 cm tall. The overall weight of the robot is approximately 2.1 kg, as presented in Figure 20.

## 4. Discussion

The design and construction of a 1:3-scale *Acinonyx jubatus* quadruped robot prototype showcase significant advancements in bio-inspired robotics. The choice of scale ensured a balance between biomechanical accuracy and the feasibility of incorporating components and materials, ultimately allowing for a faithful emulation of the *Acinonyx jubatus*’ movements. The experimental validation of the prototype movements, using image processing, provided key insights into the biomechanical design. The close correlation between the theoretical and experimental angular positions of the limb joints suggests that the kinematic model is robust and can accurately predict the physical behavior of the robot’s limbs during different gaits.

The choice of the Wilson–Cowan model over alternative neural network models is justified by the capability to coordinate complex motor movements through rhythmic patterns that are inherently synchronous. Such synchronization is critical for maintaining stability and continuity in the locomotion of quadruped robots across varied and unpredictable terrains. Furthermore, the Wilson–Cowan framework’s adaptability allows for the fine-tuning of parameters to suit specific locomotive tasks. This is critical in applications where the robot must navigate through challenging terrains, adjusting its gait in response to environmental stimuli, much like its biological counterparts. The Wilson–Cowan model provides a foundation for such adaptations, as they are derived from empirical observations of neural behavior in a wide range of species. Nevertheless, the CPG model was based on a review of the literature, where we identified a need for control strategies that extend mechanical mimicking, exploring neuromechanical synchrony. The Wilson–Cowan model has been shown to be particularly effective in capturing the essence of animal locomotion, enabling the design of robots that can execute complex movements such as pacing and galloping with more biological accuracy. The implementation of this model illustrates not only the fidelity of our prototype’s movements to natural gait patterns but also highlights the model’s utility in robotic capabilities in terms of adaptability and efficiency across various terrains. The reliability of the quadruped gait patterns to those of the *Acinonyx jubatus* may be achieved through a combination of material selection and control algorithms. The congruence between simulated and actual joint angles, as illustrated in Figure 18 and Figure 19, quantitatively supports the effectiveness of the design parameters. The use of Power HD-1501MG servomotors, with a torque output of 17 kg-cm and a swift response time of 0.14 s per 60-degree rotation, contributed to the prototype’s dynamic capabilities. Nevertheless, the use of aluminum and acrylic in the robot’s structure may offer a balance between mechanical resilience and mass efficiency, facilitating agility and strength in locomotion. However, some limitations were noted. The absence of ground interaction, such as friction forces, in the simulations suggests that further work is required to understand how the robot would perform in a real-world environment. The observed discrepancies in correlation values, particularly at the knee joint during the hindlimb movement, indicate potential areas for refinement in either the physical construction or the kinematic modeling of the robot. The implementation of Wilson–Cowan neural oscillators in the control strategy has been validated as a successful approach to mimicking natural gait patterns. Future iterations of this research may focus on enhancing the control algorithm to adjust for real-time feedback and environmental interactions, which would likely improve the adaptability and stability of the robot’s movements. Overall, the results obtained from the design, simulations, and experimental processes demonstrate promising progress towards achieving a robotic platform that can replicate the unique locomotive characteristics of the cheetah. With further development and refinement, such robots have the potential to revolutionize fields requiring rapid and flexible movement across varied terrains.

From a social impact point of view, the application of biomimetic quadruped robots in search-and-rescue operations exemplifies a significant potential beyond academic research. These kinds of quadruped robots, which are outfitted with advanced sensors and imaging technology, are designed to navigate through dangerous areas and handle unstable surfaces. This makes it possible for them to carry out essential tasks like searching through fields of destruction left behind by natural catastrophes, using thermal imaging to find missing people, and securely transporting supplies. Their design allows them to adapt to a variety of environmental circumstances, guaranteeing operational efficacy in situations where human teams are exposed to significant hazards. In this manner, these robots improve rescue operations while putting the security of human responders first [33,34]. Another relevant application in environmental monitoring is that biomimetic quadruped robots can access areas that are otherwise challenging for human researchers or traditional machinery. With the help of environmental sensors, these robots monitor animals, collect essential ecological data, and measure the quality of the air and water, all of which contribute to conservation and sustainability efforts [35,36,37].

The development of a biomimetic quadruped robot illustrates the critical importance of interdisciplinary collaboration in tackling complex technological challenges. Specialists from robotics, biomechanics, and computer science each played pivotal roles. Robotics engineers designed mechanical and electrical systems to mimic biological movements accurately, while biomechanical experts refined motion algorithms to ensure the genuine replication of natural gaits. Computer scientists developed adaptive control systems using neural networks and machine learning, enabling the robot to perform autonomously across diverse environments. This collaborative effort not only showcased the complexity required for such innovative projects but also set a precedent for future technological endeavors [36,38].

By including these applications in our discussion, we aim to raise awareness of biomimetic quadruped robots and highlight their potential uses in a variety of fields, such as environmental conservation and emergency response.

## 5. Conclusions

This paper details the design, simulation, and creation of a quadruped robot prototype inspired by the *Acinonyx jubatus* (cheetah), aimed at emulating its distinct walking, trotting, and galloping locomotive patterns. The prototype was designed with 12 DOFs after a musculoskeletal anatomy and biomechanics analysis. A bio-inspired control mechanism, employing a CPG model based on Wilson–Cowan neural oscillators, was developed for each limb, interconnected via synaptic weights, facilitating the generation of oscillatory signals that mimic four distinct gaits. The formulation of the mathematical transformation hierarchy model and the subsequent simulation of direct kinematics are also presented in this paper. Simulations of the robot’s movements were conducted in a mechanics software environment, leading to the physical realization of a prototype with dimensions of 35.5 cm in length, 21 cm in width, and 27 cm in height and an approximate weight of 2.1 kg (proportions on a 1:3 scale). The experimental validation of the prototype’s limb angular positions and trajectories, achieved through image processing techniques applied to video-captured movements, revealed correlation coefficients from 0.9025 to 0.9560 across most joint angular positions, except for the knee joint, which demonstrated a correlation of 0.7071.

The results of this study may provide new opportunities for improving robotic mobility, stability, and adaptability in the future in addition to confirming the feasibility of such bio-inspired robotic systems. This contribution to the field of robotics emphasizes the importance of bio-inspiration in the development of advanced robotic systems capable of navigating diverse environments with agility and precision akin to the biological counterparts.

## Figures and Tables

**Table 1:** State of the art in quadruped robot development: this table summarizes pivotal advancements in quadruped robotics, showcasing innovations, speed, weight, and complexity across various projects.

Year	Authors	Key Features	Speed	Weight	Degrees of Freedom
2004	Palmer and Marhefka [6].	Mammal-like limb configuration, three degrees of freedom.	25 km/h	75 kg	3
2011	Lewis M. A. et al. [7].	Back member employing biarticular muscle concept, light leg generating 90 N of force.	Not specified	Leg: 1.7 kg	Not specified
2012	Boston Dynamics [8].	Methanol combustion engine, hydraulic drive system, 14 degrees of freedom.	29 km/h	154 kg	14
2013	BioRob [9].	Lightweight, 8 degrees of freedom.	1.42 m/s	1 kg	8
2016	Steve W. Heim et al. [10].	Active tail for legged robots to simplify control via decoupling objectives.	Not specified	Not specified	Not specified
2023	Hua Chen et al. [11].	Capturability and push recovery via switched systems for dynamic balance.	Not specified	Not specified	Not specified

**Table 2:** Central pattern generator applications in robotic locomotion: this table provides an overview of key research milestones employing CPGs in the development of robotic locomotion.

Year	Authors	Locomotion	Methodology
2002	J. Shan and F. Nagashima [13].	Humanoid Biped	CPG circuit modeled by recurrent neural networks.
2007	H. Kimura, Y. Fukuoka, and A. H. Cohen [14].	Quadruped	Artificial neural system model consisting of a CPG and reflexes, reflex mechanisms, and spring mechanisms.
2010	A. Spröwitz et al. [15].	Reconfigurable	CPG as a distributed motion controller connected with certain optimization algorithms.
2011	C. Liu, Q. Chen, and D. Wang [16].	Quadruped	CPG-based 3D workspace trajectory generator and a motion engine.
2012	T. Wang et al. [17].	Biped Hopping	Two-level CPG control mechanism coupled with feedback information.

**Table 3:** Anatomical measurements of the *Acinonyx jubatus*.

Variables		Unit	Values
Intergirdle distance		mm	760 ± 10
Mass		kg	48 ± 6
Forelimb length (without scapula)		mm	670 ± 10
Hindlimb length		mm	820 ± 10
Scapula length		mm	160 ± 10
Forelimb segment without scapula (% forelimb length)	Humerus	%	34.0 ± 1.3
	Radius	%	38.8 ± 1.2
	Metacarpal	%	15.0 ± 0.7
	Phalanges	%	12.2 ± 0.3
	Femur	%	31.7 ± 0.7
Hindlimb segment (% hindlimb length)	Tibia	%	38.0 ± 0.9
	Metatarsal	%	19.2 ± 0.5
	Phalanges	%	11.1 ± 0.5

**Table 4:** Robot segments metrics (scale 1:3).

Variables		Size [mm]
Intergirdle distance (Forelimb–Hindlimb)		255
Intergirdle distance (Left–Right)		120
Forelimb length (without scapula)		225
Hindlimb length		27.5
Scapula length		55
Forelimb segment without scapula	Humerus	75
	Radius–Metacarpal	120
Hindlimb segment	Femur	90
	Tibia	105
	Metatarsal	55

## Data Availability

All data used in this research are available upon request from the corresponding author.
